# Biological Consequences of Declining Human Fertility

**DOI:** 10.1002/puh2.70207

**Published:** 2026-03-19

**Authors:** Maciej Henneberg, Frank Rühli

**Affiliations:** ^1^ Institute of Evolutionary Medicine University of Zurich Zurich Switzerland; ^2^ School of Medicine Adelaide University Adelaide Australia

## Abstract

Human fertility has been declining for over a century, especially in economically developed nations. This decline is a result of the changing socio‐economic situation that triggered the “demographic transition.” The progress in medical sciences and in living conditions has resulted in a strong reduction of infant and child mortality, which, together with increasing family economic expectations and female economic independence, has produced a situation of a few children being born. It appears that the short time over which the fertility declined and the socioeconomic causes of this decline did not allow directional forces of evolution to affect biological determinants of human reproductive abilities. It is argued here that the alteration of the mutation/selection balance over the lifetime of a few generations is sufficient to affect human ability to produce offspring—fecundity—and thus we have already entered R.Aitken's “post‐transition trap” that reduces “future fecundity of our species.” It is no longer just a hypothesis. Reduced fertility will lead to a situation in which the use of artificial reproductive technologies will become an increasingly more relied upon way to reproduce humans unless methods to remove from the gene pool deleterious mutations affecting human fecundity are developed.

## 1 Main

Fertility has declined precipitously in industrialized nations; however, it is difficult to distinguish between its reversible, mostly socioeconomic, and irreversible biological causes [1, 2]. The “post‐transition trap” hypothesis [1] postulates that in the distant future, persistent low rates of human fertility may influence our biological ability to produce offspring—fecundity—through alteration of the mutation/selection balance, thus making the human population less able to maintain its numbers. It is argued here that this process has already started and will progress rapidly.

Fertility is defined as the actual number of offspring born. It may be measured as the total fertility rate (the number of children born by a female during her lifetime) or the specific fertility—for instance, the age‐specific fertility that shows the number of offspring born per year to mothers in a given age range (e.g., 20–24 years). Data on female fertility are plentiful and fairly accurate. Male fertility is less often measured because paternity is not as certain as maternity. Fecundity is the ability of an organism to produce offspring that directly affects evolutionary fitness [3]. The notions of fertility and fecundity are often confused, for example, calling an infecund individual “infertile.”

Although fertility is easy to measure by counting the number of offspring born, measuring fecundity is difficult. As fertility is currently a joint result of biological fecundity and socio‐economically regulated birth control, both negative (contraception and abortions) and positive (assisted reproductive technologies, ARTs), it is difficult to decide whether human fecundity is changing. Although fertility can be manipulated by changing its socio‐economic determinants, fecundity cannot be manipulated this way. It is a biological property of human bodies.

The composition of the human gene pool, and thus the biological characteristics of humans, depends on the operation of the forces of evolution. There are two directional forces: mutation and selection [4]. The mutation/selection balance ultimately determines the composition of the human gene pool. Most mutations occur randomly as errors in the DNA sequences, affecting either a single nucleotide (single nucleotide polymorphisms—SNPs) or as chromosomal aberrations (translocation of a section of one chromosome onto another, deletion of a chromosome fragment, or a change in a number of chromosomes (e.g., trisomy). Some may be caused by mutagenic agents in special circumstances. The probable effect of mutations is the deterioration of the biological integrity of the structure and function of organisms [5]. The passing of mutations to the next generation is controlled by natural selection. It acts as a janitor of the gene pool, removing deleterious mutations.

Natural selection operates through the variation of the reproductive performance (Box 1). According to J. Crow [6], the opportunity for natural selection can be measured by the index *I_s_* = *P_d_*/*P_s_* + *V_f_*/*x* ^2^, where *P_d_* is the proportion of individuals dying before reaching the reproductive age, *P_s_* is the proportion of individuals surviving to the reproductive age, *V_f_* is the variance of fertility, and *x* is an average number of children born to a female during her lifetime (=total fertility rate). This index does not measure the actual intensity of selection, only its opportunity.

1 **BOX 1**

Measuring Human Reproduction

Fertility and mortality together determine human reproduction, the process in which dying individuals are replaced by newly born ones. However, terminological confusion occurs because many authors consider reproduction to be just the characteristics and activities related to fertility [7], whereas demographers and evolutionary biologists consider reproduction as the entire process of renewing population numbers through time using measures like the net reproductive rate (NRR), combining data on mortality and fertility [8]:

$\mathrm{NRR}=\sum_{0}^{\omega }b_{x}l_{x},$

Where *b_x_* is the fertility rate at age *x*, *l_x_* is the survivorship to age *x*, and *ω* is the oldest age in a population.

When generation time (*G*, the average age of parents at birth of their offspring) is varying, then a more precise measure of the overall reproductive performance is provided by the intrinsic rate of natural increase (*r*)[9]:

r=ln(NRR)/G

Natural selection occurs through differences in reproductive performance of bearers of different genetic endowments. Thus, the measure of evolutionary fitness of a group of individuals is a ratio of their intrinsic rate of natural increase to that of the best reproducing group. *W_i_* = *r_i_*/*r*_max_ [10].

The decline of population numbers occurs when the intrinsic rate of natural increase is less than zero (negative). Whether it happens due to increased mortality or decreased fertility is irrelevant. A population may die out due to low fertility even when mortality is very limited.

To measure the actual selection intensity, the heritability of reproductive performance must be known. This heritability is the portion of the total variance that is determined by the additive effects of genes. The heritability of mortality has been measured a number of times by various methods, taking into account the age at death of biological relatives [11, 12]. It is obvious that human age at death is a result of a number of factors, many of them being external to the human body; thus, the heritability, which is the portion of additive genetic variance in the total variance of the age at death, is relatively small.

Heritability of fertility is equally difficult to measure due to the influence of numerous non‐biological factors, such as rules of marriage, birth control, and environmental factors affecting the number of offspring produced. In a study of birth intervals, carefully controlling for external factors influencing fertility, it was estimated that the portion of genetic variance in the total fertility variance is small, approximately 0.15 [13].

Therefore, the actual intensity of natural selection acting on the human gene pool is many times smaller than estimates of the opportunity for selection. The intense mortality of infants, children, adolescents, and young adults was a significant factor of natural selection until recent times [14, 15, 16]. The variance of fertility, though poorly heritable, played some role in shaping the human gene pool before the Malthusian ideas of population growth disparity with the economic growth took root in industrializing populations from the 19th century onward. Although Malthus proposed only moral restraint in producing offspring, his statement that population growth must be limited to avoid a shortage of food led to the intense use of various methods of birth control. Initially, it increased the environmental (socio‐economic) variation of the number of children born to females who practiced or did not practice conscious birth control. As birth control practices became widespread, they ultimately reduced the opportunity for natural selection through differential fertility (*I_f_* = *V_f_*/*x* ^2^) because most females had only one or two children irrespective of their greater or lower biological ability to bear offspring. An example is in Table 1. It may be argued that during the demographic transition, a gradual reduction of the total fertility rates is accompanied by the transitional increase in the variation of fertility rates and in Crow's index of the opportunity for selection [17]. This possible increase of the opportunity for selection has been counterbalanced by the fact that people in the countries undergoing the demographic transition are experimenting with methods of the negative birth control. Birth control increases the variance of individual total fertility rates caused by sociocultural factors having no relationship to the variance in fecundity. In brief, genetic variance in fecundity has been reduced in modern times. Fecundity is understood here as the biological potential of reproductive output over the lifetime of a person.

**TABLE 1:** Total average fertility rates (TFR = *x*), variance (*V_x_*) of the total number of children born to a female during her lifetime, and the index of opportunity for selection (*I_f_*) in a number of populations in Poland (Henneberg 1980).

Population (or sample), date	*N*	Total fertility rate average (TFR = *x*)	Variance of individual TFRs (*V_x_*)	Opportunity for selection *I_f_* = *V_x_*/*x* ^2^
Szczepanowo, 1928–1875	73	5.49	7.87	0.26
Nur 1963	572	3.63	5.46	0.42
Wdzydze Kiszewskie 1975–1979	35	4.83	5.29	0.23
Chmielno 1975–1979	28	6.43	12.39	0.30
Gniewino 1975–1979	29	5.52	4.32	0.14
School teachers, Koło 1978	31	2.65	0.72	0.10

*Note:* Szczepanowo—a rural population 1828–1875; Nur—rural East Poland 1963; and Wdzydze Kiszewskie, Chmielno, and Gniewino—rural populations in the NE Poland studied in 1975–1979. Kolo—a small town primary school teachers 1978.

Before the beginning of the 19th century, humans practiced birth control in various forms, attempting to both increase and decrease the number of offspring. These attempts were sporadic and aimed at specific goals, such as not producing an illegitimate child or increasing the number of heirs. The number of children in families was a subject of some regulation [18, 19, 20, 21].

Specific religious communities, such as the Hutterites, tried to increase their fertility.[17] In others, simple methods of contraception (e.g., coitus interruptus or periodic abstinence) kept child numbers below the maximum possible. A simple example is the TFR of ∼6 in a 19th century rural Catholic community in Poland compared to the Hutterite TFR of ∼10 at the same time [13, 22].

The intense negative birth control became widespread in industrialized countries in the second half of the 19th century, when, with the substantial reduction in child mortality, the expenditure on child upbringing became a part of family economic considerations. It brought a practical end to any operation of natural selection on human fecundity. The number of children born per couple became primarily a result of their economic calculations, whereas the natural biological factors, including fecundity, took a second place. It can be stated that the heritability of fertility, generally low, has been reduced to near zero by largely effective birth control. Couples of low fecundity who desired a child started seeking medical help to conceive and gradually became successful in their task using an increasing number of ARTs. Their offspring possibly inherited parental genetic determinants of poor fecundity [23], so that their probability of having their own offspring became lower and dependent on ART. The only selection that remained is that of removing from the gene pool mutations causing complete infecundity [24, 25]. The word “infertility” in relation to a couple is used deliberately instead of “infecundity” because there are three causes of the complete infertility of a couple: (1) infecundity of just one or both members of the couple; (2) incompatibility of gametes that prevents the production of a zygote of this particular combination of prospective parents; or (3) a combination of parental haploid genomes that makes the embryo or early fetus unviable. De novo mutations occurring in the embryo that make it unviable cause the loss of pregnancies, but because mutations are random, they affect only single pregnancies, not the entire outcome of repeated attempts at producing a live birth.

In general, there has been a strong tendency towards relaxing the operation of natural selection on human fecundity. It is similar to the influence of relaxed natural selection on a number of other human biological characteristics (e.g., diabetes Type I, some cancers, and anatomical variations) [16, 26, 27, 28, 29, 30], but different in that it is the variation in fertility rather than the mortality that is the cause. Ultimately, it is human fecundity that is affected by the alteration of the mutation/selection balance.

Fecundity of both sexes needs to be considered. The empirical evidence for reduced male fecundity is controversial [31, 32]. The large literature on deficiencies in the sperm count, sperm motility, and sperm morphology is criticized in terms of its methods. However, the facts described in this literature are real. For whatever, perhaps methodological, reasons, the sperm count is now lower than half a century ago, and sperm characteristics are less normal than they were earlier [33].

The literature on female fecundity reduction is difficult to find systematically because various biological conditions that may reduce female fecundity are either described from the point of view of various pathologies (e.g., endometriosis, ovarian, and breast cancers) or are underlying female childlessness that, however, has many nongenetic causes. Estimates of female infertility vary.[34, 35]

Although the theoretical argument that relaxed natural selection lowers human fecundity is clear, empirical evidence is equivocal. The reason is methodological because there are various ways of measuring infertility and childlessness that reflect a greater or lesser influence of non‐biological factors. Historical demography indicates that before the advent of industrialization, about 5% of cohabiting couples were infertile or childless [36], whereas now variously measured infertility or childlessness exceeds 10%.[31, 34, 37] Thus, there is approximately a doubling of infertility, possibly infecundity, over some eight generations.

The genetics of human infertility are not well‐known yet, beyond the fact that many genes contribute to infertility in specific cases [35, 38]. Human fertility and fecundity are polyfactorial characteristics. Fecundity, an essentially biological trait, though open to the impact of many environmental factors, has a strong polygenetic component [39]. However, in contrast to many polygenic characteristics such as body height or skin color, where effects of numerous alleles add small effects to increase or decrease the whole, effects of alleles influencing fecundity may not be additive but disruptive, breaking the chain of events producing fertile gametes, viable zygotes, successful placentation, and well‐developing embryos. It suffices to break one link in the chain to reduce fecundity. Therefore, the effects of deleterious mutations will not necessarily sum up in a person, gradually lowering their fecundity; rather, each of the mutations on its own may be damaging fecundity completely. At the population level, effects of various fecundity‐affecting deleterious mutations will quickly add up to result in a large increase in childlessness of couples because it takes only one of the prospective parents to be infecund to cause the couple's infertility.

Let's assume that the frequency of each fecundity‐disrupting mutation is similar to that of common chromosomal aberrations like the chromosome 21 trisomy, about 0.001,[40] or Turner Syndrome 0.0005 [41]. The total incidence of chromosomal abnormalities reaches 0.0168 [42], and they affect males and females equally. As about 100 of de novo mutations occur in each newly formed zygote [43] and a small proportion (e.g., 0.1%) of them may be detrimental to human fecundity, it can be estimated that the rate of fecundity‐affecting mutations and chromosomal aberrations per generation is about 0.02. With the ART now allowing passage of some fecundity‐affecting genes to the next generation [23], the effect of relaxed selection will be the fast increase of biologically caused childlessness. The overall childlessness of couples is not just an effect of infecundity. It has social, economic, and personal causes. In the pre‐industrial past, especially in traditional societies such as those of peasants, however, most of the childlessness was a result of infecundity. In such rural populations, childlessness varied between 0.03 and 0.08 [13]. Taking into account pre‐industrial levels of childlessness, about 5%, future changes in childlessness caused by genetic infecundity can be estimated from the model of genetic change under relaxed selection, assuming all deleterious mutations are recessive [44]:

*C_i_*_+1_ = *C_i_* + *k^x^* (μ/s)^0.5^, where *C_i_* is the childlessness in generation *i*, *C_i_*_+1_ is the childlessness in the next generation, *k* is the number of loci involved, *μ* is the mutation rate, and *s* is the rate of selection. Using the number of loci involved, *k* = 10, mutation rate, *μ* = 10^−5^, selection rate *s*, declining by 0.1 per generation from the pre‐industrial *s* = 1.0, it can be estimated that infecundity increased from some 5% in 1900 to around 15% current magnitude (2000–2020) after four generations (of 26–30 years). It will increase to 35% in the next century (2100–2140) and 75% a century later. In 15 generations, around the year 2300, it will reach 100%. Of course, changing the number of alleles, their mutation rates, or the rates of selection can quantitatively alter results, but the fact remains that a mutatio/selection balance altered by relaxation of selection will rapidly increase childlessness.  As not all childlessness, even in pre‐industrial times, had a biological basis, these numbers may be somewhat different, but the seriousness of the situation is real.

The threat of infertility for biological reasons can be compared to the threat of an ecological catastrophe resulting from the climate change. Everybody is talking about it, but no efficient action is taken, whereas day‐to‐day human activities continue to exacerbate the situation.

Similar to the results of the relaxation of the natural selection through the differential mortality, where various mutations affecting human survival are no longer removed from the gene pool, but their effects can be treated clinically (e.g., Type I diabetes and some cancers), ensuring human survival, the relaxed selection through differential fertility may impact human fecundity to an extent requiring ART to conceive a child. Such a situation endangers the survival of humanity, not just the free choice of females and males intending to produce offspring. We are facing a situation where nearly all children will have to be artificially “created.” What would happen if ART became unavailable for some difficult‐to‐forecast reasons?

## 2 Economic Consequences

**FIGURE 1:**
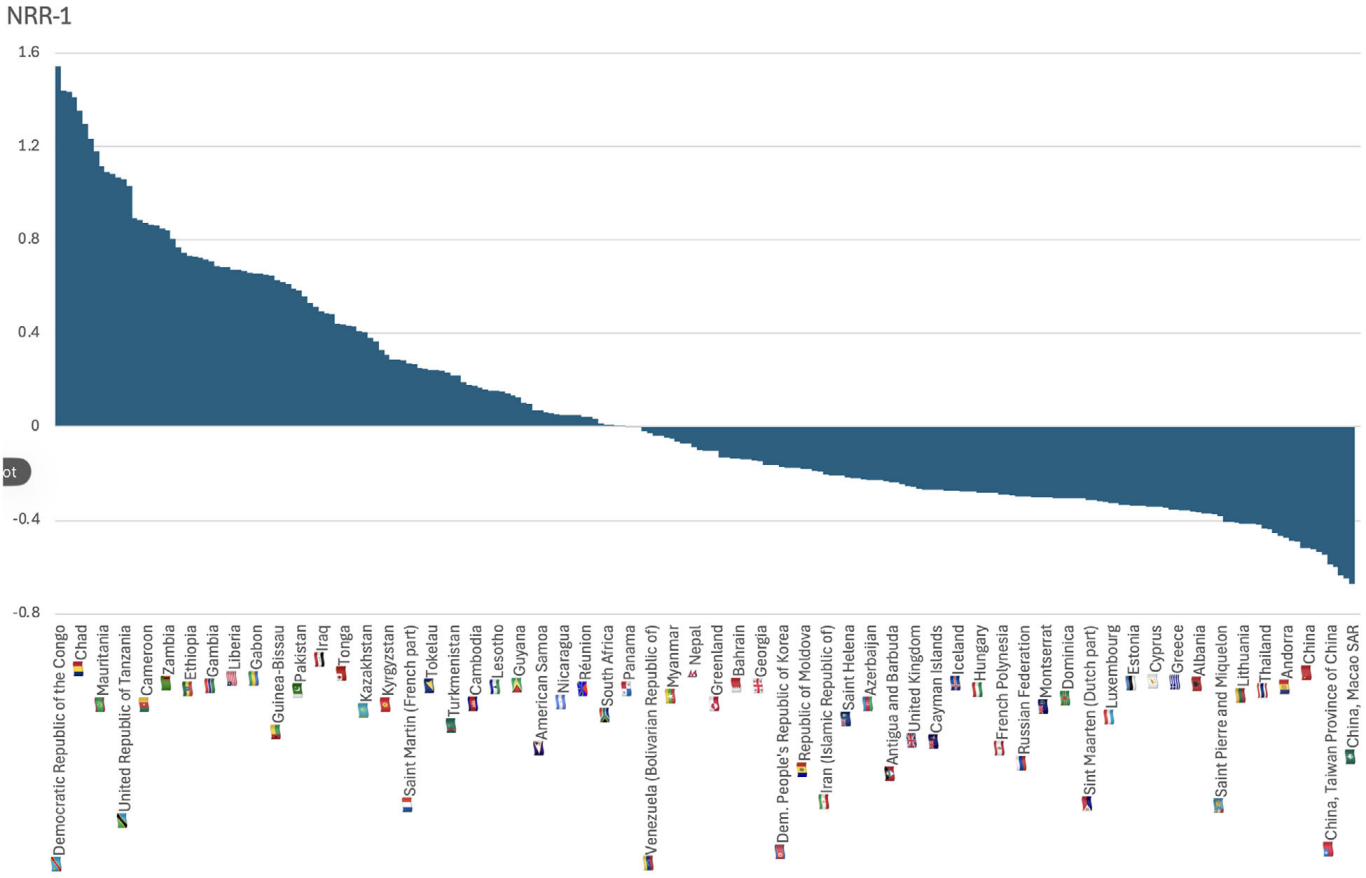
Net reproductive rates by country, 2004. Most countries of the world have a reproductive deficit. To illustrate reproductive deficit, 1 was subtracted from the actual value of the net reproductive rate derived from https://database.earth/population/net‐reproduction‐rate. NRR, net reproductive rate.

**FIGURE 2:**
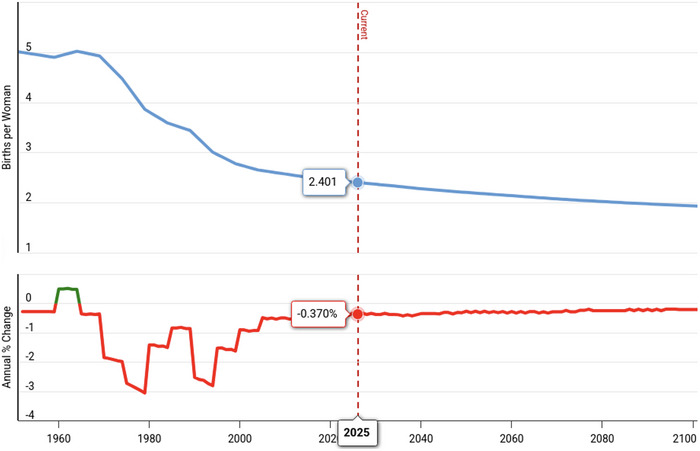
Decline in the total fertility rates worldwide. Note the prediction of continued decline towards 2100. Image from: <'https://www.macrotrends.net/global‐metrics/countries/WLD/world/fertility‐rate'> World Fertility Rate 1950‐2025. www.macrotrends.net. Retrieved 2025‐02‐03.

**FIGURE 3:**
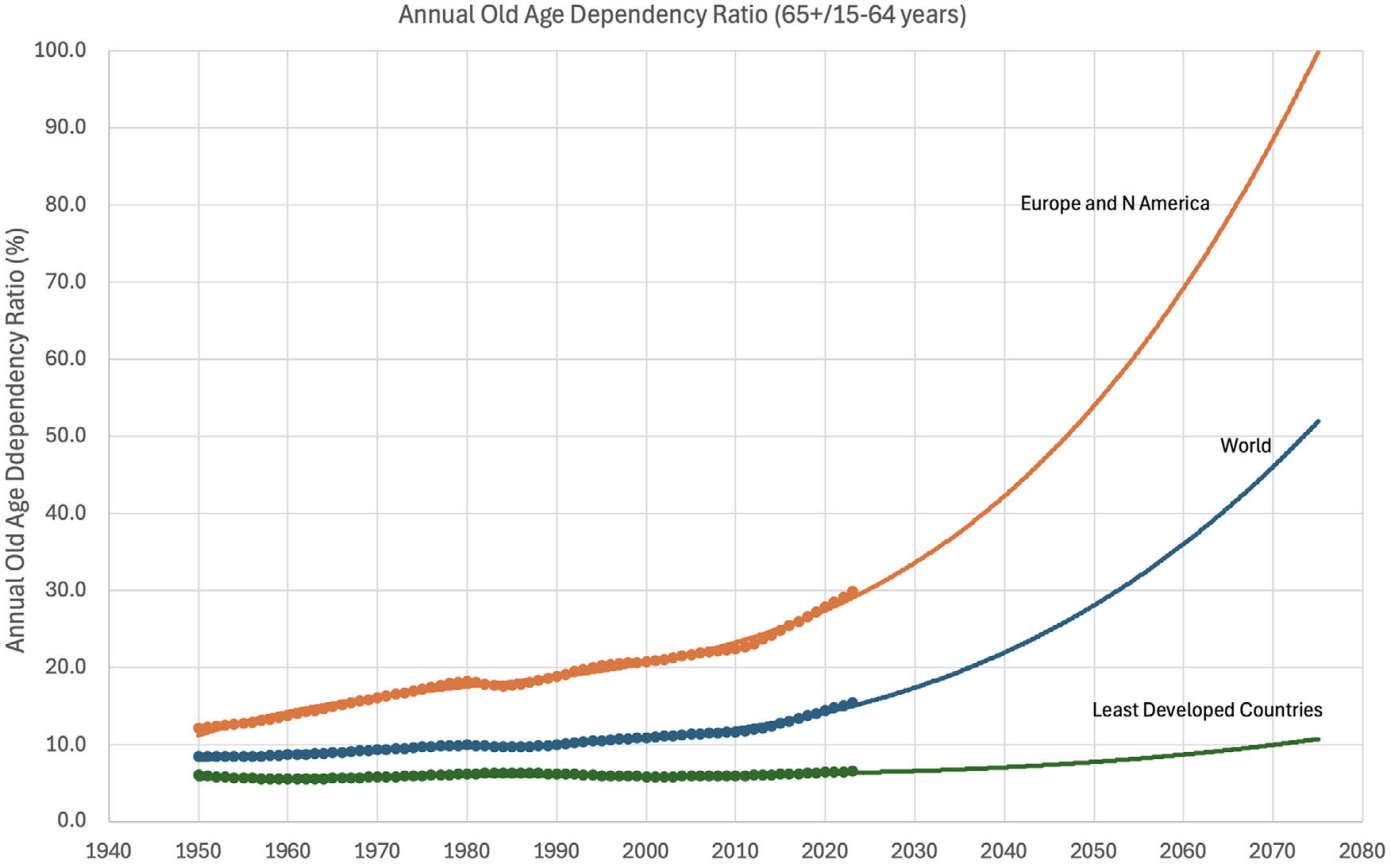
Consequences of declining fertility for human population structure—increasing dependency ratio. The dependency ratio depicted is the number of people aged 65+ as a percentage of the working‐age population (15–65 years). Data for 1950–2023 from the UN Population Council (https://population.un.org/wpp/downloads?folder = Standard%20Projections&group = Population) supplemented by own projections based on fitting the third‐degree polynomial regression lines. Note that in European and North American countries, the ratio will reach 100% around the year 2075, meaning that every working‐age person will have to support one elderly individual. Unbearable economic burden. This will happen in the lifetime of young adults alive today.

The declining fecundity may have dire demographic consequences via irreversibly lowered fertility. Already, more than half of the Earth's countries have reproductive rates below population replacement levels (Figure 1), and when total fertility rates of the world population decline to 2.0 by the end of this century (Figure 2), the decline in global population numbers will ensue. The decline in the size of a population as such may be beneficial; however, when it occurs via lowered fertility, the population age structure changes so that there are progressively lower numbers of children and young individuals, increasing the proportion of older individuals in the population. Some of those older individuals retire, becoming economically inactive, whereas the young adult segment of the population is not adequately replaced by a declining number of children who grow up. This process increases the dependency ratio—the proportion of older individuals who require the economic support of younger adults. An increasing dependency ratio has already occurred in the world, especially in more developed countries, producing a substantial burden on the working‐age population (Figure 3). This hurts the economy. The dwindling human population in the next century will have an unfavorable age structure—the population pyramid will be top‐heavy. We have to face the fecundity and fertility problem now because it takes a few generations’ time to reverse demographic demise.

## Author Contributions

Maciej Henneberg and Frank Rühli conceived the idea of this article during mutual discussions. Maciej Henneberg drafted the manuscript and the display items. Frank Rühli revised and edited the text and the display items.

## Funding

The authors have nothing to report.

## Ethics Statement

The authors have nothing to report.

## Consent

The authors have nothing to report.

## Conflicts of Interest

The authors declare no conflicts of interest.

## Data Availability

All data are publicly available in the literature and on official websites cited.
